# Comparison of online learning designs during the COVID-19 pandemic within bioinformatics courses in higher education

**DOI:** 10.1093/bioinformatics/btab304

**Published:** 2021-07-12

**Authors:** Sanna Abrahamsson, Marcela Dávila López

**Affiliations:** Bioinformatics Core Facility, University of Gothenburg, Gothenburg, 405 30, Sweden

## Abstract

**Motivation:**

Due to the worldwide COVID-19 pandemic, new strategies had to be adopted to move from classroom-based education to online education, in a very short time. The lack of time to set up these strategies, hindered a proper design of online instructions and delivery of knowledge. Bioinformatics-related training and other onsite practical education, tend to rely on extensive practice, where students and instructors have a face-to-face interaction to improve the learning outcome. For these courses to maintain their high quality when adapted as online courses, different designs need to be tested and the students’ perceptions need to be heard.

**Results:**

This study focuses on short bioinformatics-related courses for graduate students at the University of Gothenburg, Sweden, which were originally developed for onsite training. Once adapted as online courses, several modifications in their design were tested to obtain the best fitting learning strategy for the students. To improve the online learning experience, we propose a combination of: (i) short synchronized sessions, (ii) extended time for own and group practical work, (iii) recorded live lectures and (iv) increased opportunities for feedback in several formats.

**Supplementary information:**

Supplementary data are available at *Bioinformatics* online.

## 1 Introduction

The COVID-19 pandemic spread throughout the world during spring of 2020 (Cucinotta and Vanelli, 2020). In order to reduce and prevent the dissemination of the SARS-CoV-2 virus (Hellewell *et al.*, 2020), higher education around the world was forced to deliver education through online courses (UNESCO, 2020).

After almost a year, although students prefer face-to-face learning over online learning (Patricia Aguilera-Hermida, 2020), some have accepted synchronized online classes (Khalil *et al.*, 2020) and felt that online education is useful (Chakraborty *et al.*, 2020). Teachers are mastering different online platforms to deliver their class material (Bergdahl and Nouri, 2020) and are now more concerned about how to shape their material to further engage their students.

Adapting traditional onsite courses to remote learning was thought to be a temporary solution, however, with the prolonged pandemic, it is crucial to move from emergency remote teaching to an effective online education (OECD, 2020a). Additionally, remote learning has proven to promote positive aspects such as increased time for family, new activities and personal improvement (Patricia Aguilera-Hermida, 2020). There is a new perspective on the value of online options to help students and it is plausible that online learning is here to stay.

There are several studies that have recently evaluated the rapid transition to online learning and discussed the students’ and teachers’ views (Bao, 2020; Bergdahl and Nouri, 2020; Bojović *et al.*, 2020; Crawford *et al.*, 2020; Gonzalez *et al.*, 2020; Jandrić *et al.*, 2020; Mishra *et al.*, 2020; Patricia Aguilera-Hermida, 2020). However, to our knowledge, there is a lack of studies assessing different learning design options.

This study targets five short practical courses given by the Bioinformatics Core Facility, at the University of Gothenburg (https://www.gu.se/en/core-facilities/bioinformatics-bcf). These are part of the third-cycle (doctoral studies) courses catalogue that aim to cover the need for skillful researchers to perform bioinformatics analyses. The courses included are ‘Bioinformatics and Genomics’ (*BinG*) with 4.5 higher education credits (hp), ‘R programming’ (*R*), ‘Gene expression analysis using R’ (*GenExp*), ‘Next generation sequencing data analysis’ (*NGS*) with 2 hp and ‘Unix with applications to NGS data’ (*Unix*) with 1 hp. In 2013, *BinG* and *NGS* were the first courses to be implemented, followed by *Unix* in 2015, *R* in 2018 and *GenExp* in 2019. The courses have been adjusted each year according to the students’ suggestions, and newer courses follow the structure of these improved courses.

Each course admits between 10 and 20 participants, including PhD students and postdoctoral fellows. All courses are designed as onsite training, following John Dewey’s learn-by-doing theory (Felder and Brent, 1996), where one topic per week is introduced with one-hour lecture or hands-on demonstrations, followed by practical work during the rest of the day. To foster student–student interactions, different group activities are performed during the sessions, including brainstorming, think-pair-share and pair programming, among others. Students also have the opportunity to ask questions during the assigned Q&A sessions. Before introducing a new topic in the course, the teacher guides a walkthrough of the practical to clarify and unify the newly acquired knowledge. A small quiz is held, using the online tool Socrative, where the students participate anonymously. Answers are used as a real time tool and discussed together in the group to improve the students’ overall understanding. The examination is done either through a project or written reports of the practical exercises.

Due to COVID-19, these courses were adapted to an exclusive online format. Since the courses were given consecutively, each following course was modified considering the student’s course evaluations, to meet their needs and preferences. This exploratory study aims to determine the most suitable learning design for online short practical courses within bioinformatics, to improve the graduate students learning experience.

## 2 Methodology

### 2.1 Course selection

Twelve different doctoral courses have been developed at the Bioinformatics Core Facility since 2013. Five of these courses, including *BinG*, *R*, *GenExp*, *Unix* and *NGS*, have been given annually in that order and thus were chosen to be adapted to online format. Course evaluations regarding the overall organization of the course as well as the quality and quantity of the teaching material for these courses, are summarized in Supplementary Table S1.

For the last four years (2017–2020), most of the topics were given by the same teaching staff. Exceptions are shown in Supplementary Table S2. These include topics that were given by external staff at some point during the courses. For the *Unix* course, we do not collect topic-specific feedback. In the case of the 2020 *BinG* course, topic-specific feedback was not collected since this course was adjusted to an online format during the progression of the course. We focused on the students’ overall perception of the course rather than on specific topics, since half of the topics were face-to-face and the others were online.

### 2.2 Survey design

We prepared five similar surveys, one for each given course, with 12, 14, 13, 9 and 13 questions, respectively. Each survey had a quantitative and qualitative section.

The quantitative section had four general questions, enquiring about the overall organization of the course, its content quality and quantity, the workload corresponding to the credits awarded and the communication with the course organizer and teachers. We included four-to-five course specific-questions, which focused on the overall delivery of each covered topic. All these items were divided on a 5-point Likert scale where a score of ‘1’ represented ‘Poor’ and a score of ‘5’ represented ‘Excellent’. A summary of the course evaluations is shown in Supplementary Tables S1 and S2.

In the qualitative section, there were four-to-seven open questions about how to improve the course as well as the overall design of the online course. Questions are presented below in the context of the analysis.

### 2.3 Study participants and data collection

A total of 75 students participated in the courses (39 males and 36 females) where the attendance distribution was 27%, 27%, 15%, 17% and 15% to the respective courses. Half of the students attended only one of the online courses, almost one-third attended two courses, while the rest (five students) attended three courses. No student enrolled in both versions of the same course. All students were invited to answer the corresponding anonymous, online survey at the end of each course.

We received 65 completed surveys (32 males and 33 females) where the mean age was 30.3 (SD = 4.4). These corresponded to 100%, 65%, 100%, 92% and 82% of the total amount of students of each course, respectively.

### 2.4 Data analysis

Qualitative data were analyzed for thematic content using the standard content analysis framework (Erlingsson and Brysiewicz, 2017). Data were coded and categorized through an inductive process, and relevant themes were identified by grouping these categories. Several refinement iterations were conducted until a consensus between the authors was reached.

Statistical analysis of the quantitative data was performed by applying the unpaired Wilcoxon rank sum test, where the alternative hypothesis tests that the distribution of the online courses’ median is significantly lower than the face-to-face courses. For comparisons among face-to-face courses, the alternative hypothesis tests that the distribution of the most recent course median is significantly higher than the previous course. Multiple testing correction was performed using the Benjamini–Hochberg procedure.

## 3 Adapting the course design

### 3.1 Short synchronized sessions improve concentration and understanding

The overall design of the onsite courses demand, for each topic, one entire day of traditional face-to-face activities (6–8 h). It includes one-hour lecture, that introduces the selected topic and the rest is dedicated to computer-based exercises. The teachers are available during the practical exercises to aid the students in a personalized way. This setting is valued by the students as illustrated by this quote: ‘*I like that the course is very practical (i.e. short lectures and mostly exercises)*’.

During the first adaptation to online courses, the same format and length were kept. Due to a lack of habit, the students and teachers were not able to sit for so many hours in front of the computer and maintain their focus. Therefore, for later courses, we adjusted the synchronized time by reducing it to 3 h; one-hour lecture and 2 h for initiating the practical work. Several small breaks were integrated along the session to retain the students’ concentration (Davies and Parasuraman, 1982).

### 3.2 Extended self-study time increases the students’ autonomy

To compensate the synchronized time reduction, the self-study time was increased twofold to allow the students to complete the exercises on their own, to mimic the real-life situation of analyzing their own data. Since the students decide how to allocate their time, other activities can be scheduled *ad hoc*. This gives them some freedom to work at their own pace, as pointed out by this quote: ‘*The course has a nice format with introduction on Mon. and WT. on Fri. and the freedom to choose self when to work with the* *assignements. The weekly* *assignement* *is* *great and it is good that the due day are on* *monday* *(to have that extra time if you have a very busy lab week) [sic].*’

This autonomy puts high demands on the students’ time management and prioritization skills, however, being at a graduate level, they are expected to be familiar with this environment (Gustafsson *et al.*, 2003). In agreement, 78.9% of the students reported that ‘*At first it was not* [easy to prioritize] *… but later with planning it became easy*’, just within a couple of weeks of the course adjustments.

### 3.3 Live lectures promote knowledge transfer and recording them help students to revisit the acquired knowledge

In the beginning of the pandemic, the online communication networks faced an unprecedented demand (OECD, 2020b) that affected the quality and reliability of the teaching platforms. The instability of these tools, in particular Zoom (opted tool to be used in our courses), led us to avoid live lectures. As a quick response, for some lectures (*BinG*) the script of the lecture was distributed while for other sessions (*BinG* and *GenExp*) we recorded the lecture and shared them through our internal learning platform. Fortunately, the online platforms were able to rise to the occasion and we were able to have synchronized online lectures.

**Fig. 1.**
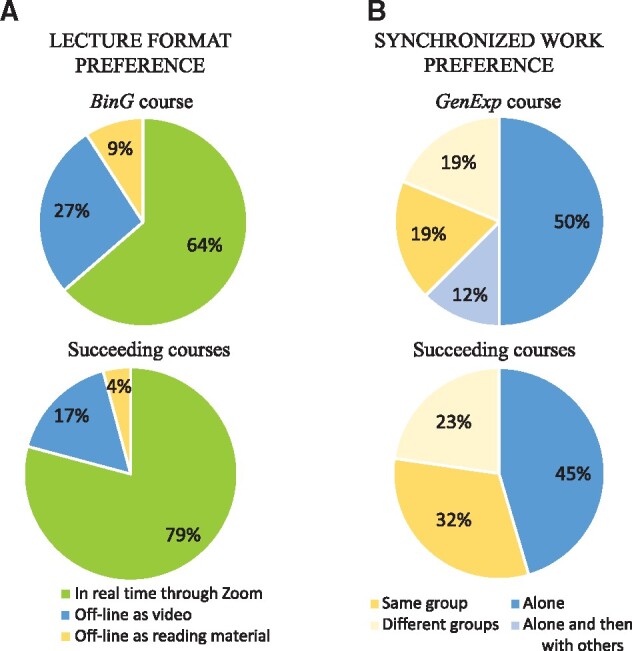
Graduate students’ preferences for short online courses. (**A**) Distribution of the students’ preference for the format of the lecture given. *BinG* was the first course adapted to its online counterpart. Succeeding courses include *R*, *Unix* and *NGS* (*GenExp* data not available). In both cases, the majority of the students preferred to have live lectures through a virtual environment (green). Around a quarter favored prerecorded lectures (blue) while the rest preferred to read the script associated with the lecture slides (yellow). (**B**) Distribution of the students’ preference for the type of group work during the assignments. *GenExp* was the first online course given during autumn. Succeeding courses include *GenExp*, *Unix* and *NGS* (autumn courses). Dark blue shows a preference for working by one-self, where light blue shows students preferring to discuss with colleagues afterwards the assignment. Yellow shows a preference of working in groups, where half of the students preferred working with the same group during the entire duration of the course (dark yellow), and the other half, preferred to interact with different course members (light yellow)

For the first online adapted course (*BinG*), only 9% of the students favored the scripts associated with the lecture slides, while almost a third preferred prerecorded lectures (Fig. 1A). Despite both, scripts and prerecorded lectures being available as notes that the students can revisit when needed, these materials lack the live interaction between student and teacher. For instance, if the student needs further explanation of a critical concept, he/she may invest a lot of time trying to understand it, where as if the lecture were live, the trainer could clarify the concept directly in a live lecture. It could also result in the loss of valuable knowledge, since there is no knowledge sharing among the group.

It is clear that the majority of the students (64%) preferred lectures in real time (Fig. 1A), which were also recorded and made available at the internal learning platform. Some students mentioned that ‘*recording the sessions was also a big relief because I could go back and watch the instructions many times*’ and ‘*uploading* [the recorded lectures] *could be nice if you miss one due to conflicting schedules*’.

Considering this information, we decided to exclude the lecture scripts for future courses. This reduced the teachers’ preparation time to transcribe the presentation notes. When looking at succeeding courses (*R*, *Unix* and *NGS*) it is clearer that real time lectures need to be kept (Fig. 1A) and that recording of such sessions are helpful, as indicated by some students: ‘*The live sessions combined with access to the lecture videos/slides allowed me to work through the material at a pace that was comfortable for me*’ and that ‘*recorded sessions could help me to resolve my problems.*’

### 3.4 Increased opportunities for feedback boost the students’ confidence about the concepts learned

In terms of the overall experience with the conversion of the first course (*BinG*) as an online alternative, 68.4% of the participants agreed that ‘*it worked rather smoothly*’. However, 21% mentioned that more interaction was needed. In response to this, we added two Q&A sessions during each week for the following courses. These sessions were optional and were attended by students (i) in need of help from the trainers to complete the assignment, (ii) wanted to try what they have learned on their data or (iii) simply wanted to have some company while working on the exercises.

We also introduced a more detailed walkthrough. These sessions covered the practical exercises, where the teacher besides discussing the answer to the exercises, thoroughly explained the data analysis procedure and the code, when applicable.

At the end of each topic, we kept the small quiz using Socrative as used with the face-to-face version of the courses.

Students mentioned that ‘*The Q&A sessions, and overall instructor accessibility, has been especially advantageous*’, that ‘*the walk throughs [sic] were valuable so that you can check if you did it correctly and get the opportunity to ask questions if you did it in another way*’, and that ‘*the* *socrative* *at the beginning of each lesson was a great addition*’, especially because it is ‘*good to see if you are keeping up*’. These sessions, in addition to helping the students, are an opportunity for the teacher to unify the group’s knowledge and review any gaps or misunderstandings.

### 3.5 Group work fosters networking

Typically, students tend to gather and discuss the practical exercises during the face-to-face version of the courses (Paechter and Maier, 2010). This behavior promotes peer-to-peer learning (Kemp and Grieve, 2014) and establishes new connections that may be fruitful in the future. As online communication tools are still being accepted in the education sector, these interactions are challenging to foster (Bassoppo-Moyo, 2006).

To deal with this bottleneck, we tested three grouping strategies during the practical exercises of four different courses. We used the breakout rooms feature from Zoom to create private study groups to apply these grouping strategies. In the first strategy, we assigned the working group and kept the same members throughout the entire *R* course. In the second strategy, the aim was to increase the communication among the students, thus every week we assigned different working groups for the *GenExp* course. Finally, for the *Unix* and the *NGS* courses, we gave the students the choice of either forming a working group of their choice to work in a breakout room or to work alone, either in the main session or in their own breakout room.

The preferences on how the students wanted to work during the practical exercises are shown in Figure 1B. For the first course given in Autumn, *GenExp*, most of the students (62%) seem to prefer working alone, since ‘*there was very little communication in the breakout rooms*’, and some students ’*need quiet and no distractions*’ to complete the exercises, besides it ‘*allows you to try and figure out how to do the different steps by yourself which I think is really important*’. Unfortunately, we do not possess this information from the face-to-face courses. Thus, we are not able to conclude whether preferring working alone, is a characteristic of students interested in learning different aspects within Bioinformatics or that, due to the high tempo in their research activities, the students can organize their study time without having to synchronize their time with a third party.

It is also worth noting that the high percentage of students that prefer working alone may be a result of targeted activities. For instance, programming courses such as *R* and *Unix*, may be prone to isolate students since learning how to program tend to be an individual process (Pea and Kurland, 1984). However, in these courses, we have adapted paired programming (Williams and Upchurch, 2001), where one writes the code and others brainstorm. The students’ high acceptance of this approach (85% for the *R* course), confirm that we must adopt activities to encourage student–student interactions.

Another point of view to consider is the technological aspect. The low number of students that are inclined to work in groups, either with the same members (19%) or different members each time (19%), could be due to the handling of a new technological platform. As the online communication tools become a crucial part within teaching and learning, these interactions will be easier to encourage and achieve in the near future (Dhawan, 2020). Indeed, this is corroborated by these students’ comments in the succeeding courses during autumn: ‘*Since these days every one [sic] is familiar with zoom app properties especially remote control, so students can help each other to learn course material in breakout rooms*’ and ‘*I was more active in discussions in later courses. I think in the beginning it was unfamiliar and people were more shy [sic]*’. Moreover, we can already observe how the preference of working in groups increases from 38% to 55% in the autumn courses (Fig. 1B), where it is likely that students that have been isolated for several months, look forward to connect with people, as pointed out by a student, ‘*working with a* *‘team’* *that stays the same over the course could be fun, but with Covid making it already hard to get to know fellow students, mixing groups could also be fun’*.

Regardless of the unprecedented circumstances that the educators and students face today, it is essential that the teaching staff contribute with the creation of meeting arenas to support peer-to-peer learning as well as scientific collaboration and networking.

### 3.6 Online courses alongside face-to-face courses

#### 3.6.1 Organization, quality and quantity

To evaluate the quality of the online courses, we compared the students’ perceptions in terms of the overall organization of the courses (including the course material, the schedule, among others), their quality (in terms of the material provided) and their quantity (in relation to the course workload), see Table 1.

**Table 1.** Overall course satisfaction survey

Course (*n*, *m*)	Category^a^	Online (2020)		Face-to-face (2019)		FDR^b^
		M	SD	M	SD	
*BinG* (19, 15)	Organization	4.58	0.61	4.40	0.83	0.992
	Quality	4.32	0.82	3.80	0.56	0.992
	Quantity	4.53	0.61	4.13	0.64	0.992
*R* (13, 16)	Organization	4.31	0.48	4.56	0.73	0.104
	Quality	4.31	0.75	4.06	0.73	0.870
	Quantity	3.23	0.73	4.13	0.73	0.010^*^
*Unix* (11, 7)	Organization	4.73	0.47	4.71	0.76	0.066
	Quality	4.82	0.40	5.00	0.00	0.066
	Quantity	3.45	1.04	3.43	0.53	0.066
*GenExp* (12, 7)	Organization	4.58	0.51	4.57	0.53	0.809
	Quality	4.42	0.51	4.57	0.53	0.809
	Quantity	4.50	0.52	3.43	0.53	0.999
*NGS* (9, 4)	Organization	4.44	0.53	5.00	0.00	0.519
	Quality	4.22	0.44	4.75	0.50	0.420
	Quantity	3.89	0.60	4.50	0.58	0.519

*n*, *m*: number of received answers in 2020 and 2019, respectively.

*BinG*: Bioinformatics in Genomics course, *R*: R programming course, *Unix*: Unix with applications to NGS data course, *GenExp*: gene expression analysis using R, *NGS*: next generation sequencing data analysis.

* Significant at FDR < 0.05 (Wilcoxon test).

a Organization includes course material, schedule, facility, information, etc.

b *P*-values are shown in Supplementary Table S3.

The online courses organization ranged from 4.31 to 4.73, where the best marking is 5 corresponding to ‘Excellent’. There was no significant difference to the face-to-face courses from 2019 which ranged from 4.40 to 5.00.

The quality was also maintained despite the different delivery methods. The online courses scored between 4.22 and 4.82, while the face-to-face courses scored between 3.80 and 5.00.

An interesting aspect was the course workload. Most of the online courses, ranging from 3.23 to 4.53, did not show any difference in comparison with face-to-face courses, which ranged from 3.43 to 4.50. The only significant discrepancy was found in the *R* course (FDR = 0.01). The course was kept unmodified while moved to its online version, since it took place close to the pandemic outbreak. It is obvious from the students’ comments that they wanted more in-depth information, affecting the rating of the course. For instance: ‘*Maybe add extra tips for more complicated commands*’, ‘*More info on other packages perhaps*’, ‘*Also, going into the functional aspects of syntaxes*’ and ‘*Give more examples*’.

With these suggestions, we revised succeeding courses and modified the practical work in the *NGS* course to improve the students’ understanding throughout the course. We also replaced the question-based format of the assignment with a detailed essay format. In response to these changes, a third of the students expressed that ‘*the workload far exceeded 2 points*’, however this did not affect the overall rating of the course after multiple testing correction (Supplementary Table S3). This illustrates the subjectivity of the course attendees where different backgrounds and skills-set define the attitude and perception of the course organization and material.

Moreover, the comparison between the student’s scoring of the face-to-face courses (2017–2019) showed no statistical difference in the organization of these courses nor in their quality or workload (Supplementary Table S4). This may suggest that the selected courses are stable and well implemented. Certainly, there is always room for improvement and our courses are not the exception.

#### 3.6.2 Experienced teaching staff eases the transition to online teaching

Since 2017, the same teaching staff has been involved in the design and delivery of the courses within this study. Considering that the same person is responsible for a specific topic over the years, we assume that any variation in the students’ scoring of the individual topics reflect the experience of the responsible teacher(s).

To evaluate in more detail the quality of the online courses, we compared the students’ perceptions for each topic included in the different courses (Table 2). One exception is the *Unix* course, where topic-wise evaluations have not been included since the course was first implemented. The second exception is the online version of the *BinG* course. We omitted the evaluation of the topic-specific feedback, given that this course was adjusted to its online counterpart during the progression of the course. The feedback would be highly biased due to the circumstances.

**Table 2.** Topic-specific satisfaction survey

Course (*n*, *m*)	Category^a^	Online (2020)		Face-to-face (2019)		FDR^b^
		M	SD	M	SD	
*R* (13, 16)	Introduction to R	4.62	0.51	4.56	0.73	0.667
	Experimental design	4.23	0.83	3.69	0.79	0.960
	Statistical analysis	4.00	1.22	3.81	1.05	0.667
	Data visualization	4.54	0.52	4.75	0.45	0.505
*GenExp* (12, 7)	qPCR	3.75	0.75	3.86	0.69	0.747
	RNAseq	4.67	0.49	4.29	0.76	0.888
	scRNAseq	4.67	0.49	4.43	0.53	0.888
*NGS* (9, 4)	QC and mapping	4.22	0.67	5.00	0.00	0.038^*^
	Targeted resequencing	4.33	0.50	4.75	0.50	0.133
	Cancer genomics	4.00	0.71	4.25	0.50	0.297
	RNAseq	4.11	0.60	5.00	0.00	0.026^*^
	Functional genomics	3.44	1.13	5.00	0.00	0.026^*^

*n*, *m*: number of received answers in 2020 and 2019, respectively.

* Significant at FDR < 0.05 (Wilcoxon test).

a Topics introduced at the different courses, given by the same instructor(s) over both courses.

b *P*-values are shown in Supplementary Table S5.

As for the *R* and *GenExp*, the overall satisfaction score of their different topics ranged between 3.44 and 4.67. There was no significant difference to their face-to-face version from 2019, ranging from 3.69 to 5.00.

As mentioned previously, we changed the practical work and the examination format in the *NGS* course. This adjustment was counterproductive and students voiced their negative opinion regarding the heavy workload during the course, as pointed by these quotes ‘*the assignments were more work than I anticipated*’ and ‘*the time it took me …* [to complete the assignments] *… doubled my workload*’. As expected, this was reflected on the scoring of the changed topics (QCandMapping, RNAseq and FunctionalGenomics), which ranged from 3.44 to 4.22 in comparison to the face-to-face format where they scored 5.00. It is undeniable that the assessment is subjective and that these discrepancies might be due to the groups’ differences rather than the content of each topic. However, since the results align with the adjusted topics, we agree on the veracity of the evaluation and will adapt the content in the next issue of the course.

Furthermore, we also compared the individual topics’ scoring between the face-to-face courses. These showed no statistical difference (Supplementary Table S6). This may suggest that the teaching staff delivers the content of each topic in a consistent manner. On the other hand, this may be an unexpected result, since it is common sense that increasing the teacher’s experience should improve the teacher’s evaluation. As aforementioned, one reason could be due to the groups’ diversity (i.e. background knowledge, motivation, technology prone, among others) rather the actual teacher’s growth.

Nevertheless, in our case, having experienced teaching staff involved in the transition of our courses to online format meant resources were easily directed toward the technological challenges without affecting the quality of the courses as we have shown in this study.

#### 3.6.3 Minor changes in the course design of well-structured courses may be enough

To assess if similar, i.e. minor changes, or substantial changes have been implemented in other Swedish institutions, we consulted the National Bioinformatics Infrastructure Sweden (https://nbis.se/), an organization that also provides courses within bioinformatics in a similar fashion as the courses presented here.

Typically, their courses are one-week full time with small lectures and practical work. During 2020, some planned courses were postponed due to the pandemic and the short time to adapt the courses online.

Two courses that were adapted to their online version were ‘*Introduction to bioinformatics using NGS data*’ and ‘*Single cell RNAseq analysis*’, which have been running for over 4 years as onsite courses. These courses were delivered via Zoom and most of their original schedule of 8 h for five consecutive days was maintained. The main change was to ensure that the students had enough breaks, while their main challenge was to promote interaction among the participants. The rating of these courses was very good, 4.7 out of 5 points for the ‘*Introduction to bioinformatics using NGS data*’ course, while 75% of the participants of the ‘*Single cell RNAseq analysis*’ course thought it was good with an online format (*personal communication*).

These findings indicate that, similarly to our online courses, no substantial changes were critical in this first attempt of adapting well established courses to an online setting. While, this may also be the case for recently organized face-to-face courses, it is undeniable that the process may be smoother when tested structure and material is available.

Nevertheless, it may be that in the near future, more notable changes will be implemented in the design of online courses that could benefit both teachers and students.

## 4 Proposed course design for short online courses at the graduate level

Overall, our newly adapted online courses were well received by the students. It cannot be discarded that the students could be biased in their assessment i.e. being more sympathetic toward our efforts to promptly adjust the courses during the pandemic. However, we are convinced that having a strong face-to-face course design and experienced teachers, laid the foundation to ease the transition toward online teaching and kept the quality of the course despite the technological challenges. Moreover, the wide range of activities within the face-to-face courses seemed to align to the online structure. As a student commented: ‘*I find the core components of lecture + recording of lecture + assignment + Q&A + Walkthrough* *to be an excellent combination.*’

**Fig. 2.**
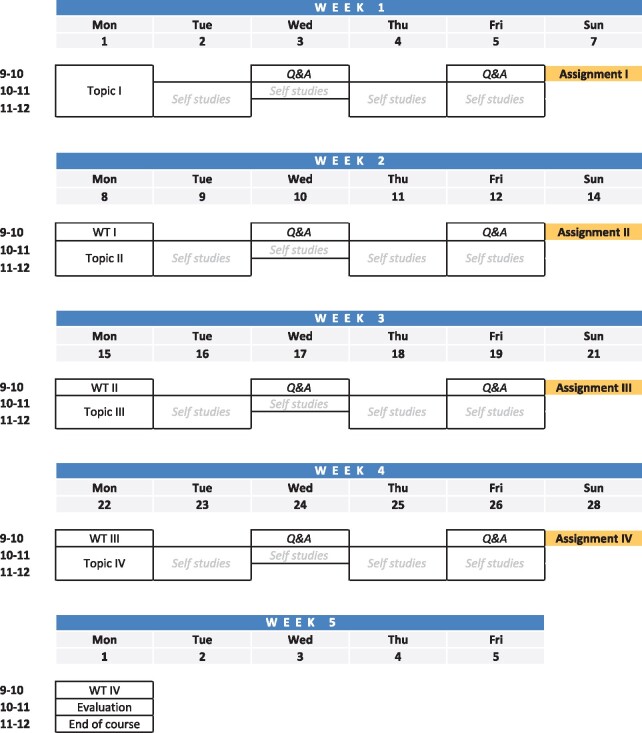
Proposed course design. Example of a course schedule for a short (2.0 HP) online course in higher education. One topic per week is revised with short lectures and an introduction to the exercises. The expected own practical work (self-studies) although clearly specified, can be adapted by each student according to their needs. There are two optional sessions for questions during the week (Q&A) and a walkthrough (WT) of the ongoing exercises. Assignments are handed in by the end of each week. The course ends with a course evaluation

We propose a basic structure design for short online courses, based on the students’ preferences (Fig. 2). In general, we propose to embrace one topic per week, giving time to the student to organize their working time as they see fit and short introductory lectures with group exercises as well as complementary hands-on assignments to be submitted by the end of the week. This will give the student feedback on his/her performance and clarify any doubt before moving toward the next topic. In addition, we propose several point checks, such as Q&A sessions, to provide guidance to the student, and detailed walkthroughs of the exercises to reinforce and homogenize the knowledge of the group. Finally, an evaluation of the course, encouraging the students to openly participate in developing a better course is crucial to design.

We foresee that this design will ease this unprecedented transition to online teaching, for both students and teachers. Although the changes proposed in this study are not strikingly significant compared to the face-to-face course design, it is relevant to note that some of these courses have been running for 7 years and have been yearly revised and adjusted considering previous student’s feedback. Furthermore, they have been used as reference for more recent developed courses.

## 5 Conclusion

This study explored different designs for short doctoral courses that were adapted to an online version, due to COVID-19. We conducted a survey to know the opinion of graduate students, attending the online version of five bioinformatics courses, with respect to their face-to-face counterparts.

The findings showed that students appreciate:

- short and synchronized sessions, where lectures are recorded and made available for future access,
- the opportunity of allocating their study time as they see fit, and
- multiple channels to corroborate their learning

In our case, this evaluation has opened an alternative approach toward teaching. On the teachers’ side, it requires (∼50%) less onsite time since the responsibility has been shifted toward the student. We are able to include students from other geographical regions, that otherwise would not be able to attend due to travel related issues. As for the students, it also creates an opportunity to improve their computer skills and feel comfortable while managing different platforms. We create a real-life environment, where students need to balance the completion of the course and their own research work. Moreover, with a less intense layout, they can decide when to focus on the course which improves their efficiency. Therefore, we have revised our courses and readjusted them to follow the course design in Figure 2. However, considering the student’s feedback, an ideal scenario that we may examine is a mixed course design where onsite and online sessions are blended to further improve the student’s experience.

In this case study, the online education was equally evaluated as their face-to-face version. This was possible due to the constant involvement of the students’ opinions in the improvement of the courses. Although these findings cannot be generalized, and more research is certainly needed, to select the best strategies that suits the different online learning characteristics of the students, these experiences may serve as a starting point while adopting online learning in higher education.

## Supplementary Material

btab304_Supplementary_Data
